# Biological characteristics and immune responses of NK Cells in commonly used experimental mouse models

**DOI:** 10.3389/fimmu.2024.1478323

**Published:** 2024-11-19

**Authors:** Jingwen Qin, Zhaokai Zhang, Haopeng Cui, Jinhua Yang, Aiqun Liu

**Affiliations:** ^1^ Department of Gastroenterology and Respiratory Internal Medicine & Endoscopy Center, Guangxi Medical University Cancer Hospital, Nanning, China; ^2^ Department of General Surgery II, Cenxi People’s Hospital, Wuzhou, Guangxi, China

**Keywords:** laboratory mice, biological characteristics, surface receptors, flow cytometry, natural killer cells (NK cells)

## Abstract

The biology of natural killer (NK) cells in commonly used mouse models is discussed in this review, along with their crucial function in a variety of immunological responses. It has been demonstrated that the formation, maturation, subtype variety, and immunological recognition mechanisms of NK cells from various mice strains exhibit notable differences. These variations shed light on the intricacy of NK cell function and offer crucial information regarding their possible uses in treating human illnesses. The application of flow cytometry in mouse NK cell research is also covered in the article. Improved knowledge of the biology of NK cells across species may facilitate the development of new NK cell-based therapeutic approaches.

## 1 Introduction

An integral part of the immune system, natural killer (NK) cells are vital for both antiviral and anti-tumor defense. Ever since their initial identification in 1975 (1), researchers studying biology and immunology have been interested in the distinct roles and modes of action of NK cells (2–4). These cells can eliminate aberrant tissue cells while preserving the body’s tolerance for healthy tissue cells as the presence of various activating and inhibitory receptors on their surface (5, 6). The potential of NK cells in immunotherapy has grown in recent years due to a growing understanding of their basic characteristics (7).

Mice share a high degree of genetic homology with humans and are easy to manipulate and breed, making them an ideal model for studying disease mechanisms (8). Laboratory mouse models play a crucial role in NK cell research. However, there are significant differences in development, subsets, and surface receptor expression between mouse and human NK cells, such as the absence of CD56 expression in mouse NK cells (9, 10), which limits the direct applicability of mouse models to human studies. Additionally, significant phenotypic and functional differences exist among NK cells from various mouse strains (11, 12). Therefore, a comprehensive understanding of the biological characteristics of NK cells in different mouse models is essential for translating basic research findings into clinical applications.

This review presents a thorough examination of the biological attributes of NK cells within frequently employed experimental mouse models, along with their efficacy across various experimental paradigms (**Table 1**). By dissecting the ontogeny, maturation, heterogeneity of NK cells, and their immune recognition processes within these models, we aspire to provide a comprehensive understanding of the role of NK cells in immunological research. This review is intended to serve as a reference for forthcoming studies in the field.

**Table 1:** Comparative characteristics of NK Cells in commonly used mouse models.

Mouse Model	NK Cell Characteristics	Development/maturation Changes	Other Related Characteristics	Cytotoxicity	References
C57BL/6	High interferon production and complement activity	Decreased number of mature NK cells with age	Ly49H positive NK cells exhibit genetic resistance to MCMV	Strong	(6, 13–27)
BALB/c	High IFN-γ expression, yet insufficient to offset NK cell functional deficiency	Not mentioned	High Th2 immune response; Absence of Ly49H expression; Susceptible to MCMV	Weaker	(28–35)
BALB/c Nude	Impaired NK cell function	Activity increases with age	Lack of thymus and T lymphocytes	Age-related	(36–40)
SCID	Profuse IFN-γ production; NK cell function is unaffected	Immune function may recover with age	NK cells confer protective effects against neurological diseases	Strong	(41–45)
NOD/SCID	Impaired NK cell function, both innate and adaptive immune deficiencies	Not mentioned	Immature NK cells abundant at fetomaternal interface	Partially impaired	(43, 46–49)
C3H/He	Significant fluctuation in NK cell proportion and activity with age	Peaks at 6 to 8 weeks, then sharply declines	Prone to mammary tumors	Age-dependent	(50, 51)
ICR	Activity and number related to rearing environment and exercise	Related to rearing environment and exercise	Strong adaptability and rapid growth	Variable	(52–56)

## 2 Biological characteristics of NK cells in commonly used experimental mouse models

### 2.1 NK cells in C57BL/6 mice

C57BL/6 mice are widely utilized in biomedical research due to their genetic homogeneity and stability, making them an excellent model for investigating the biological properties of NK cells (13). These mice are characterized by robust interferon production and complement activity, which provide advantages for investigating NK cell immune responses (14, 15). However, the C57BL/6 strain is also known to induce immune tolerance, which may impact the long-term functionality and stability of NK cells (11, 12).

The functionality of NK cells in C57BL/6 mice tends to become dysregulated with advancing age. Studies suggest that mature NK cell counts in the bone marrow, spleen, and blood of older C57BL/6 mice are much lower than those of younger counterparts, increasing their vulnerability to the mousepox virus (16). Additionally, the NK cell phenotype in aged mice undergoes changes, with decreased expression of maturity-associated markers such as CD43, CD11b, KLRG1, CD62L, and Ly6C, an increase in the expression of immaturity-associated markers, including CD27 and NKG2AEC, among others (17), potentially impacting their immune functions. Also, aging alters the number and phenotype of NK cells that dwell in the liver as well as the expression of collagen-binding integrins in conventional NK cells (18), which are pivotal for NK cell migration, tissue positioning, and the liver’s immune microenvironment.

#### 2.1.1 Ly49 family in C57BL/6 mouse NK cells

The Ly49 family of mouse NK cells is functionally analogous to human killer immunoglobulin-like receptors (KIRs) in that both regulate NK cell activity through interactions with major histocompatibility complex class I (MHC-I) molecules. However, they differ in their gene and protein structures (9, 19, 20). Ly49H is a distinctive activating receptor on C57BL/6 mouse NK cells, capable of recognizing and binding to specific molecules on the surface of infected cells, thus triggering an effective antiviral response (21, 22). Ly49H is crucial not only for antiviral activities but also for genetic resistance to murine cytomegalovirus (MCMV) (6, 23–25). Ly49H-positive NK cells utilize mitochondrial-associated proteins BNIP3 and BNIP3L to recognize and clear dysfunctional mitochondria, enhancing the survival capacity of antigen-specific NK cells induced by MCMV (26).

Recent studies suggest that the expression of Ly49 receptors is not random but follows a specific differentiation trajectory, indicating a pattern in NK cell population differentiation. The surface expression of Ly49I is considered a pivotal step in NK cell maturation, further influencing their functional state (27).

### 2.2 NK cells in BALB/c mice

BALB/c mice exhibit a higher liver-to-body weight and spleen-to-body weight ratio compared to other inbred mice, which correlates with their pronounced Th2 immune response characteristics (28). Although the innate immune system of BALB/c mice contributes to infection clearance, its efficacy is limited and often relies on adaptive immune responses (6, 29, 30).

#### 2.2.1 “Immune deficiency” of BALB/c mouse NK cells

Research indicates that there are notable differences in CD11c expression patterns between NK cells and specific immune cells in BALB/c mice, potentially affecting their immune response to viral infections (31). Unlike C57BL/6 mouse NK cells, which resist MCMV infection due to Ly49H receptor expression, BALB/c mouse NK cells lack Ly49H, exhibiting reduced cytotoxicity and more severe symptoms upon MCMV infection (6). Despite elevated interferon-gamma (IFN-γ) expression, BALB/c mice cannot fully compensate for the NK cell functional deficiency, which may contribute to their increased susceptibility to MCMV (32).

Despite these immune shortcomings, BALB/c mouse NK cells still contribute to infection resistance (33, 34). Moreover, BALB/c mouse NK cells may also be implicated in the antidepressant effects by modulating the release of inflammatory factors secreted by macrophages (35).

### 2.3 NK cells in mutant mouse strains

#### 2.3.1 BALB/c nude mice

The BALB/c nude mouse, first developed in 1966, is a mouse model that exhibits significant immune dysfunction due to a mutation in the Foxn1 gene, which is distinguished by the absence of a thymus and T lymphocytes (36, 37). In nude mice, this mutation causes poor development, reduced fertility, and increased vulnerability to infection. However, B lymphocytes and NK cells continue to function in some capacity in BALB/c nude mice (38). Investigation reveals that NK cell activity in these nude mice is age-related, with lower activity at 3 to 4 weeks of age and increased activity by 6 to 8 weeks of age (39), which may be related to the maturation and functional development of NK cells.

Further research has revealed the complexity of NK cell activity in BALB/c nude mice. A study by Manďáková et al. found that 3-acetylpyridine, a neurotoxin, significantly increased cytotoxic activity in splenic NK cells of BALB/c nude mice (40). This indicates that even in the context of immune deficiency, the basic immune mechanisms of NK cells are regulated by innate and extrinsic factors.

#### 2.3.2 Severe combined immunodeficiency mice

In 1983, Bosma first described in detail a mouse model with severe combined immunodeficiency (SCID), which lacks functional T and B lymphocytes. Despite appearing similar to normal mice, SCID mice have significantly underdeveloped thymi, spleens, and lymph nodes, with weights typically less than one-third of those of normal mice, showing clear deficiencies in cellular and humoral immune functions (41). The function of NK cells remains unaffected in SCID mice, providing a unique perspective for investigating the role of these cells within the immune system (42, 43). In fact, some SCID mice may exhibit a degree of immune function recovery with age (44), a phenomenon that is not yet fully understood. Additionally, despite the lack of adaptive immune responses in SCID mice, the role of NK cells in these mice should not be overlooked. Investigation has indicated that NK cells in SCID mice can produce large amounts of IFN-γ and play an important role in the protection against neurological diseases (44).

Additional research has revealed that during pregnancy, SCID mice’s NK cells have a particular pattern of development. Hiyama et al. demonstrated that the absence of functioning T and B cells may cause the development of NK cells to be delayed in the early stages of the placenta in SCID mice that are pregnant (45).

#### 2.3.3 NOD/SCID mice

The non-obese diabetic (NOD) mouse serves as a model for diabetes caused by aberrant T-lymphocyte infiltration and pancreatic beta cell loss (57). In addition to diabetes, NOD mice show a number of immunodeficiencies, such as a lack of NK cells and reduced complement and macrophage activity (58). The NOD/SCID mice were generated from a cross between NOD mice and SCID mice, incorporating immunodeficiency traits from both, including the absence of T and B cells, decreased NK cell function, both innate and adaptive immunodeficiencies, and a loss of haemolytic complement activity (46, 47). These features have made NOD/SCID mice a popular model for studies involving NK cell insufficiency.

However, recent studies have found that while NK cell activity is compromised in NOD/SCID mice, it is not entirely eliminated (43). A study by Miao et al. showed that there was no discernible difference in the percentage of splenic NK cells between NOD/SCID mice and CB17/SCID mice (a model with normal NK cells but absent B and T lymphocyte function), indicating that NOD/SCID mice’s NK cell function is only partially impaired (48). Therefore, care should be taken while employing NOD/SCID mice as NK cell deficiency models.

Additionally, studies have found that in pregnant NOD/SCID mice, there is a large number of immature NK cells at the fetomaternal interface, which are insensitive to Toll-like receptor (TLR) agonist stimulation, potentially contributing to the maintenance of immune tolerance during pregnancy (49).

### 2.4 NK cells in other mouse models

#### 2.4.1 C3H/He mice

The C3H/He mouse strain originated from the crossbreeding of albino Bagg female mice with DBA male mice, which are prone to mammary tumors, followed by inbreeding. Investigations have indicated that the counts and activity of NK cells in the liver of C3H/He mice fluctuate significantly with age, emerging at 4 weeks, peaking between 6 and 8 weeks, and then declining sharply after 9 weeks (50, 51).

#### 2.4.2 ICR mice

ICR mice were developed by Hauschka within the Swiss mouse lineage, targeting high fertility (52). Celebrated for their adaptability, rapid growth, and experimental reproducibility, ICR mice are extensively utilized in pharmacological, oncological, and immunopharmacological screenings, as well as in pathological model replications (53, 54). Petitto et al. discovered that ICR mouse strains with varying aggressive behaviors display differences in cellular immune responses, with those showing less aggression having reduced NK and T cell activity and an increased likelihood of tumor development (55). Furthermore, it has been shown that both male and female ICR mice can escalate blood NK cell counts with an enriched environment and exercise, especially under group housing conditions, where the impact on male NK cells is more pronounced (56).

## 3 Molecular and functional characteristics of mouse NK cells

### 3.1 Developmental and maturation markers of NK cells

NK cell maturation is marked by shifts in surface marker expression, pivotal for identifying developmental stages. While human NK cells use CD56 and CD16 levels to denote maturation from CD56^bright^ CD16^-^ to CD56^dim^ CD16^bright^, mouse NK cells employ CD27 and CD11 (59, 60). Immature mouse NK cells are distinguished by low CD11b and high CD27 expression(CD11b^low^CD27^high^), transitioning to double-positive status(CD27 ^+^CD11b^+^), and culminating in mature NK cells with low CD27 and high CD11b expression(CD27^low^ CD11b^high^) (61). This maturation is intrinsically linked to the acquisition of effector functions.

The CD27^-^CD11b^+^ and CD27^+^CD11b^-^subsets in mice correspond functionally to theCD56^dim^ and CD56^brigh^ subsets in humans (62), aiding cross-species understanding of NK cell roles in immune surveillance and response.

### 3.2 Diversity of NK cell subsets

Mouse NK cell subset distribution mirrors human diversity, varying across tissues and peripheral blood (62). Notably, in C57BL/6 and BALB/c mice, lung lymphocyte NK frequencies surpass those in other tissues, often presenting a more mature phenotype. Pulmonary NK cells in mice are critical for sustaining immune balance, with a high proportion of mature phenotype NK cells under steady-state conditions (63). In C57BL/6 mice, most pulmonary NK cells exhibit the phenotype of CD11b^high^CD27^low^,indicating that they might be important for lung immune responses (64).

The CD11c^+^B220^+^NKcell subset in C57BL/6 mice is particularly cytotoxic and secretes IFN-γ, playing a vital role in tumor cell killing and MCMV resistance (31, 65). Liver-derived CD11c^+^B220^+^ NK cells also curb pulmonary tumor metastasis by IFN-γ secretion and modulation of the fibrinogen deposition microenvironment (66).

### 3.3 Balance of NK cell activating and inhibitory receptors

Mouse NK cells, as innate immune cells, express a range of surface receptors, including Ly49, NKR-P1, and CD94/NKG2 family members, central to NK cell immune responses (21, 67, 68). NK cell function is modulated by activating receptors and the equilibrium of inhibitory receptors, with decreased activating receptor expression potentially leading to NK cell dysfunction (69). It is noteworthy that human and mouse NK cells express activating and inhibitory receptors in quite different ways. Key activating receptors in mouse NK cells include NKp46, Ly49H, DNAM-1, NKG2D, and NK1.1, while inhibitory receptors include NKG2A and Ly49C, among others (9, 19, 70). Similar to human NK cells, a decrease in the expression of activating receptors can lead to NK cell dysfunction. NKG2D is one of the key activating receptors in both human and mouse NK cells. In mouse NK cells, NKG2D can directly trigger cytotoxic responses, whereas in human NK cells, NKG2D typically requires the cooperation of additional signals (71). This indicates that the function of NKG2D in mouse NK cells may be more autonomous. Although the cytotoxic effect of DNAM-1 in mouse NK cells is relatively weak, it contributes positively to antiviral and antitumor activities. DNAM-1 not only promotes the clearance of virus-infected cells mediated by NK cells but, when absent, may also increase the risk of tumor cell metastasis (72, 73). Chan et al. found that mice lacking DNAM-1 expression exhibit a significantly increased rate of melanoma metastasis (74). Furthermore, the overexpression of inhibitory receptors can also lead to NK cell dysfunction. J. Wang et al. found that pulmonary NK cells in mice typically exhibit higher inhibitory receptors compared to splenic NK cells. In contrast, they have relatively lower levels of activating receptors. Consequently, the activation of lung NK cells must overcome a greater threshold of inhibition (64). However, research on the differences in activating and inhibitory receptors among various mouse models remains relatively limited.

Receptor diversity on NK cells also dictates responses to various tumors, with the C57BL/6 model showing consistent NK cell responses to different tumor types, suggesting a non-specific, patterned response (75).

## 4 Immune recognition of mouse NK cells

Flow cytometry (FCM) is a vital tool in immunology for identifying and separating NK cell subsets. Despite commonalities with human NK cells, functionally analogous subset identification across species is complex. Human NK cell-specific molecules like CD56 and certain activating/inhibitory receptors are not present in mice. Researchers often use NK1.1, NKp46, and CD49b to identify mouse NK cells (76).

NKp46, part of the natural cytotoxicity receptors (NCRs), is considered a pan-NK cell marker due to its broad expression across mammalian NK cells. However, NKp30 and NKp44 are absent in mice (77), complicating specific NK cell identification. There are also strain-specific differences in NK cell surface marker expression; for instance, C57BL/6 and SJL mice use NK1.1 for identification, whereas BALB/c mice, due to allelic variations, rely on CD49b and NKp46 in the absence of NK1.1 responsiveness (76, 78).

Accurate NK cell analysis in flow cytometry hinges on selecting the appropriate antibodies. **Table 2** provides a list of recommended antibodies for mouse NK cell staining (76), aiding researchers in precise NK cell subset identification and differentiation.

**Table 2:** List of currently recommended antibodies for surface and intracellular staining of mouse NK Cells.

Sources	Marker	Format	Final Concentration	Clone	Function
BD Biosciences	CD3	PE CF594	0.2µg/test	145-2C11	T cell marker
	CD45.2	AlexaFlu700	0.1µg/test	104	Leukocyte common antigen
	CD19	PE CF594	0.1µg/test	1D3	B cell marker
	NK1.1	BV510	0.4µg/test	PK136	NK cell activation marker in certain mouse strains
	CD49a	AlexaFluor647	0.05µg/test	Ha31/8	Used to distinguish CD49b^-^CD49a^+^ ILC1s
	CD11b	BV510	0.05µg/test	M1/70	Used to identify the stages of NK cell maturation
	NKp46	BV421	0.4µg/test	29A1.4	Activation receptor, specific for NK cells
	granzyme B	PE	5μl/test	GB11	Cytotoxic granule component
eBiosciences	NKp46	PerCP-eFluor710	0.4µg/test	29A1.4	
	CD49b	PE-Cy7	0.1µg/test	DX5	NK cell marker in certain mouse strains
	Eomes	APC	0.2µg/test	Dan11mag	Associated with mature NK cells
Biolegend	CD19	APCCy7	0.1µg/test	6D5	
	NKp46	APC	0.4µg/test	29A1.4	
	NK1.1	PE-Cy7	0.4µg/test	PK136	
	CD3	FITC	0.5µg/test	145-2C11	
	IFN-γ	BV421	0.2µg/test	XMG1.2	Cytokine
	CD19	FITC	0.5µg/test	6D5	

A test is defined as the amount (μg) of antibody required to stain a cell sample in a final volume of 100 μL, with the cell count ranging from 10^5^ to 10^8^ cells per test. ILC: innate lymphoid cell.

## 5 Summary and prospects

In scientific research, the robustness, reliability, and reproducibility of experimental data are of paramount importance. To enhance the reproducibility of studies and minimize bias to the greatest extent possible, researchers must possess a comprehensive understanding of the potential phenotypic differences among mouse models and select models that align with their specific research objectives. Investigating the biology of NK cells in different mouse models serves as a crucial tool for elucidating the role of these cells in diverse immune responses. This review aims to provide reference guidelines for studies involving NK-mediated immunity assays, assisting researchers in selecting the most suitable models to address specific scientific questions. We hope that an in-depth study of the properties of NK cells in these models will establish a more robust scientific foundation for the development of NK cell-based treatment strategies for human diseases, particularly in the fields of infectious disease and cancer therapy.

## References

[1] Kiessling R Klein E Wigzell H. Natural” killer cells in the mouse. I. Cytotoxic cells with specificity for mouse Moloney leukemia cells. Specificity and distribution according to genotype. Eur J Immunol. (1975) 5:112–7. doi: 10.1002/eji.1830050208 1234049

[2] Kondo M Weissman IL Akashi K. Identification of clonogenic common lymphoid progenitors in mouse bone marrow. Cell. (1997) 91:661–72. doi: 10.1016/s0092-8674(00)80453-5 9393859

[3] Jamieson AM Isnard P Dorfman JR Coles MC Raulet DH. Turnover and proliferation of NK cells in steady state and lymphopenic conditions. J Immunol. (2004) 172:864–70. doi: 10.4049/jimmunol.172.2.864 14707057

[4] Orr MT Lanier LL. Natural killer cell education and tolerance. Cell. (2010) 142:847–56. doi: 10.1016/j.cell.2010.08.031 PMC2945212 20850008

[5] Kim N Choi J-W Park H-R Kim I Kim HS. Amphotericin B, an anti-fungal medication, directly increases the cytotoxicity of NK cells. Int J Mol Sci. (2017) 18:1262. doi: 10.3390/ijms18061262 28608807 PMC5486084

[6] Brown MG Dokun AO Heusel JW Smith HR Beckman DL Blattenberger EA. Vital involvement of a natural killer cell activation receptor in resistance to viral infection. Science. (2001) 292:934–7. doi: 10.1126/science.1060042 11340207

[7] Vivier E Rebuffet L Narni-Mancinelli E Cornen S Igarashi RY Fantin VR. Natural killer cell therapies. Nature. (2024) 626:727–36. doi: 10.1038/s41586-023-06945-1 38383621

[8] Mouse Genome Sequencing Consortium Waterston RH Lindblad-Toh K Birney E Rogers J Abril JF. Initial sequencing and comparative analysis of the mouse genome. Nature. (2002) 420:520–62. doi: 10.1038/nature01262 12466850

[9] Colucci F Caligiuri MA Di Santo JP. What does it take to make a natural killer?, Nature Reviews. Immunology. (2003) 3(5):413–25. doi: 10.1038/nri1088 12766763

[10] Hayakawa Y Huntington ND Nutt SL Smyth MJ. Functional subsets of mouse natural killer cells. Immunol Rev. (2006) 214:47–55. doi: 10.1111/j.1600-065X.2006.00454.x 17100875

[11] Mekada K Yoshiki A. Substrains matter in phenotyping of C57BL/6 mice. Exp Anim. (2021) 70:145–60. doi: 10.1538/expanim.20-0158 PMC8150240 33441510

[12] Abdel Aziz N Berkiks I Mosala P Brombacher TM Brombacher F. Environmental and microbial factors influence affective and cognitive behavior in C57BL/6 sub-strains. Front Immunol. (2023) 14:1139913. doi: 10.3389/fimmu.2023.1139913 37180163 PMC10166845

[13] Stough JMA Dearth SP Denny JE LeCleir GR Schmidt NW Campagna SR. Functional characteristics of the gut microbiome in C57BL/6 mice differentially susceptible to plasmodium yoelii. Front Microbiol. (2016) 7:1520. doi: 10.3389/fmicb.2016.01520 27729904 PMC5037233

[14] Brand A Singer K Koehl GE Kolitzus M Schoenhammer G Thiel A. LDHA-associated lactic acid production blunts tumor immunosurveillance by T and NK cells. Cell Metab. (2016) 24:657–71. doi: 10.1016/j.cmet.2016.08.011 27641098

[15] Orange JS Wang B Terhorst C Biron CA. Requirement for natural killer cell-produced interferon gamma in defense against murine cytomegalovirus infection and enhancement of this defense pathway by interleukin 12 administration. J Exp Med. (1995) 182:1045–56. doi: 10.1084/jem.182.4.1045 PMC2192290 7561678

[16] Fang M Roscoe F Sigal LJ. Age-dependent susceptibility to a viral disease due to decreased natural killer cell numbers and trafficking. J Exp Med. (2010) 207:2369–81. doi: 10.1084/jem.20100282 PMC2964566 20876312

[17] Beli E Duriancik DM Clinthorne JF Lee T Kim S Gardner EM. Natural killer cell development and maturation in aged mice. Mech Ageing Dev. (2014) 135:33–40. doi: 10.1016/j.mad.2013.11.007 24361677 PMC4043280

[18] Nair S Fang M Sigal LJ. The natural killer cell dysfunction of aged mice is due to the bone marrow stroma and is not restored by IL-15/IL-15Rα treatment. Aging Cell. (2015) 14:180–90. doi: 10.1111/acel.12291 PMC4364830 25399821

[19] Forbes CA Scalzo AA Degli-Esposti MA Coudert JD. Ly49C-dependent control of MCMV Infection by NK cells is cis-regulated by MHC Class I molecules. PloS Pathog. (2014) 10(5):e1004161. doi: 10.1371/journal.ppat.1004161 24873973 PMC4038614

[20] Zhang X Feng J Chen S Yang H Dong Z. Synergized regulation of NK cell education by NKG2A and specific Ly49 family members. Nat Commun. (2019) 10:5010. doi: 10.1038/s41467-019-13032-5 31676749 PMC6825122

[21] Smith HRC Heusel JW Mehta IK Kim S Dorner BG Naidenko OV. Recognition of a virus-encoded ligand by a natural killer cell activation receptor. Proc Natl Acad Sci USA. (2002) 99:8826–31. doi: 10.1073/pnas.092258599 PMC124383 12060703

[22] Zeleznjak J Popovic B Krmpotic A Jonjic S Lisnic VJ. Mouse cytomegalovirus encoded immunoevasins and evolution of Ly49 receptors - Sidekicks or enemies? Immunol Lett. (2017) 189:40–7. doi: 10.1016/j.imlet.2017.04.007 28414184

[23] Daniels KA Devora G Lai WC O’Donnell CL Bennett M Welsh RM. Murine cytomegalovirus is regulated by a discrete subset of natural killer cells reactive with monoclonal antibody to Ly49H. J Exp Med. (2001) 194:29–44. doi: 10.1084/jem.194.1.29 11435470 PMC2193438

[24] Lee SH Girard S Macina D Busà M Zafer A Belouchi A. Susceptibility to mouse cytomegalovirus is associated with deletion of an activating natural killer cell receptor of the C-type lectin superfamily. Nat Genet. (2001) 28:42–5. doi: 10.1038/ng0501-42 11326273

[25] Parikh BA Piersma SJ Pak-Wittel MA Yang L Schreiber RD Yokoyama WM. Dual requirement of cytokine and activation receptor triggering for cytotoxic control of murine cytomegalovirus by NK cells. PloS Pathog. (2015) 11:e1005323. doi: 10.1371/journal.ppat.1005323 26720279 PMC4697817

[26] O’Sullivan TE Johnson LR Kang HH Sun JC. BNIP3- and BNIP3L-mediated mitophagy promotes the generation of natural killer cell memory. Immunity. (2015) 43:331–42. doi: 10.1016/j.immuni.2015.07.012 PMC5737626 26253785

[27] Millan AJ Hom BA Libang JB Sindi S Manilay JO. Evidence for prescribed NK cell ly-49 developmental pathways in mice. J Immunol. (2021) 206:1215–27. doi: 10.4049/jimmunol.2000613 33495236

[28] Watanabe H Numata K Ito T Takagi K Matsukawa A. Innate immune response in Th1- and Th2-dominant mouse strains. Shock. (2004) 22:460–6. doi: 10.1097/01.shk.0000142249.08135.e9 15489639

[29] Arase H Mocarski ES Campbell AE Hill AB Lanier LL. Direct recognition of cytomegalovirus by activating and inhibitory NK cell receptors. Science. (2002) 296:1323–6. doi: 10.1126/science.1070884 11950999

[30] Scalzo AA Yokoyama WM. Cmv1 and natural killer cell responses to murine cytomegalovirus infection. Curr Top Microbiol Immunol. (2008) 321:101–22. doi: 10.1007/978-3-540-75203-5_5 18727489

[31] Liao Y Liu X Huang Y Huang H Lu Y Zhang Y. Expression pattern of CD11c on lung immune cells after disseminated murine cytomegalovirus infection. Virol J. (2017) 14:132. doi: 10.1186/s12985-017-0801-x 28720115 PMC5516330

[32] Lu Y Liu X Huang Y Liao Y Xi T Zhang Y. The effects of IL10 and NK cells on the susceptibility to mouse cytomegalovirus in BALB/c mice despite the compensation of IFNγ. Intervirology. (2018) 61:111–22. doi: 10.1159/000493316 30336455

[33] Lenac T Arapović J Traven L Krmpotić A Jonjić S. Murine cytomegalovirus regulation of NKG2D ligands. Med Microbiol Immunol. (2008) 197:159–66. doi: 10.1007/s00430-008-0080-7 18259774

[34] Lodoen MB Lanier LL. Viral modulation of NK cell immunity. Nat Rev Microbiol. (2005) 3:59–69. doi: 10.1038/nrmicro1066 15608700

[35] Hu L Li D Ge C Liao H Wang Y Xu H. Natural killer cells may exert antidepressant-like effects in mice by controlling the release of inflammatory factors. Neuroscience. (2019) 401:59–72. doi: 10.1016/j.neuroscience.2019.01.002 30641114

[36] Flanagan SP. Nude”, a new hairless gene with pleiotropic effects in the mouse. Genet Res. (1966) 8:295–309. doi: 10.1017/s0016672300010168 5980117

[37] Nehls M Pfeifer D Schorpp M Boehm T. New member of the winged-helix protein family disrupted in mouse and rat nude mutations. Nature. (1994) 372(6501):103–7. doi: 10.1038/372103a0 7969402

[38] Pan B Wei X Xu X. Patient-derived xenograft models in hepatopancreatobiliary cancer. Cancer Cell Int. (2022) 22:41. doi: 10.1186/s12935-022-02454-9 35090441 PMC8796540

[39] Qin C Zhang L Wei H. Experimental Animal Science, in: Experimental Animal Science. Beijing: People’s Medical Publishing House (2010). p. 42.

[40] Manďáková P Červinková M Virtová M Šíma P. NK cytotoxic activity and relative distribution of NK cells in 3-acetylpyridine influenced mice. Folia Microbiol. (2001) 46:173–4. doi: 10.1007/BF02873599 11501408

[41] Bosma GC Custer RP Bosma MJ. A severe combined immunodeficiency mutation in the mouse. Nature. (1983) 301:527–30. doi: 10.1038/301527a0 6823332

[42] Bodhankar S Woolard MD Sun X Simecka JW. NK cells interfere with the generation of resistance against mycoplasma respiratory infection following nasal-pulmonary immunization. J Immunol. (2009) 183:2622–31. doi: 10.4049/jimmunol.0802180 PMC2811958 19625649

[43] Okada S Vaeteewoottacharn K Kariya R. Application of highly immunocompromised mice for the establishment of patient-derived xenograft (PDX) models. Cells. (2019) 8:889. doi: 10.3390/cells8080889 31412684 PMC6721637

[44] Sellon DC Knowles DP Greiner EC Long MT Hines MT Hochstatter T. Depletion of natural killer cells does not result in neurologic disease due to Sarcocystis neurona in mice with severe combined immunodeficiency. J Parasitol. (2004) 90:782–8. doi: 10.1645/GE-205R 15357069

[45] Hiyama M Kusakabe KT Kuwahara A Wakitani S Khan H Kiso Y. Differentiation of uterine natural killer cells in pregnant SCID (scid/scid) mice. J Vet Med Sci. (2011) 73:1337–40. doi: 10.1292/jvms.11-0189 21628864

[46] Tyagi RK Tandel N Deshpande R Engelman RW Patel SD Tyagi P. Humanized mice are instrumental to the study of plasmodium falciparum infection. Front Immunol. (2018) 9:2550. doi: 10.3389/fimmu.2018.02550 30631319 PMC6315153

[47] Yong KSM Her Z Chen Q. Humanized mice as unique tools for human-specific studies. Arch Immunol Ther Exp (Warsz). (2018) 66:245–66. doi: 10.1007/s00005-018-0506-x PMC6061174 29411049

[48] Miao M Masengere H Yu G Shan F. Reevaluation of NOD/SCID mice as NK cell-deficient models. BioMed Res Int. (2021) 2021:8851986. doi: 10.1155/2021/8851986 34805408 PMC8598338

[49] Lin Y Zhong Y Saito S Chen Y Shen W Di J. Characterization of natural killer cells in nonobese diabetic/severely compromised immunodeficient mice during pregnancy. Fertil Steril. (2009) 91:2676–86. doi: 10.1016/j.fertnstert.2007.08.087 18410933

[50] Itoh H Abo T Sugawara S Kanno A Kumagai K. Age-related variation in the proportion and activity of murine liver natural killer cells and their cytotoxicity against regenerating hepatocytes. J Immunol. (1988) 141:315–23. doi: 10.4049/jimmunol.141.1.315 3379309

[51] Crow JF. C. C. Little, cancer and inbred mice. Genetics. (2002) 161:1357–61. doi: 10.1093/genetics/161.4.1357 PMC1462216 12196385

[52] Kim J Ekram MB Kim H Faisal M Frey WD Huang JM. Imprinting control region (ICR) of the Peg3 domain. Hum Mol Genet. (2012) 21:2677–87. doi: 10.1093/hmg/dds092 PMC3363340 22394678

[53] Kim J. Multiple YY1 and CTCF binding sites in imprinting control regions. Epigenetics. (2008) 3:115–8. doi: 10.4161/epi.3.3.6176 18458536

[54] Li X-Y Zhao Y Sun M-G Shi J-F Ju R-J Zhang C-X. Multifunctional liposomes loaded with paclitaxel and artemether for treatment of invasive brain glioma. Biomaterials. (2014) 35:5591–604. doi: 10.1016/j.biomaterials.2014.03.049 24726749

[55] Petitto JM Gariepy JL Gendreau PL Rodriguiz R Lewis MH Lysle DT. Differences in NK cell function in mice bred for high and low aggression: genetic linkage between complex behavioral and immunological traits? Brain Behav Immun. (1999) 13:175–86. doi: 10.1006/brbi.1998.0539 10373280

[56] Nakamura Y Ueno A Nunomura Y Nakagaki K Takeda S Suzuki K. Effects of inducing exercise on growing mice by means of three-dimensional structure in rearing environment. Exp Anim. (2016) 65:403–11. doi: 10.1538/expanim.16-0025 PMC5111843 27301720

[57] Makino S Kunimoto K Muraoka Y Mizushima Y Katagiri K Tochino Y. Breeding of a non-obese, diabetic strain of mice. Jikken Dobutsu. (1980) 29:1–13. doi: 10.1538/expanim1978.29.1_1 6995140

[58] Kikutani H Makino S. The murine autoimmune diabetes model: NOD and related strains. Adv Immunol. (1992) 51:285–322. doi: 10.1016/s0065-2776(08)60490-3 1323922

[59] Quatrini L Della Chiesa M Sivori S Mingari MC Pende D Moretta L. Human NK cells, their receptors and function. Eur J Immunol. (2021) 51:1566–79. doi: 10.1002/eji.202049028 PMC9292411 33899224

[60] Del Zotto G Antonini F Pesce S Moretta F Moretta L Marcenaro E. Comprehensive phenotyping of human PB NK cells by flow cytometry. Cytometry A. (2020) 97:891–9. doi: 10.1002/cyto.a.24001 32198974

[61] Chiossone L Chaix J Fuseri N Roth C Vivier E Walzer T. Maturation of mouse NK cells is a 4-stage developmental program. Blood. (2009) 113:5488–96. doi: 10.1182/blood-2008-10-187179 19234143

[62] Crinier A Milpied P Escalière B Piperoglou C Galluso J Balsamo A. High-dimensional single-cell analysis identifies organ-specific signatures and conserved NK cell subsets in humans and mice. Immunity. (2018) 49:971–986.e5. doi: 10.1016/j.immuni.2018.09.009 30413361 PMC6269138

[63] Zhang H He F Li P Hardwidge PR Li N Peng Y. The role of innate immunity in pulmonary infections. BioMed Res Int. (2021) 2021:6646071. doi: 10.1155/2021/6646071 33553427 PMC7847335

[64] Wang J Li F Zheng M Sun R Wei H Tian Z. Lung natural killer cells in mice: phenotype and response to respiratory infection. Immunology. (2012) 137:37–47. doi: 10.1111/j.1365-2567.2012.03607.x 22612500 PMC3449245

[65] Taieb J Chaput N Ménard C Apetoh L Ullrich E Bonmort M. A novel dendritic cell subset involved in tumor immunosurveillance. Nat Med. (2006) 12:214–9. doi: 10.1038/nm1356 16444265

[66] Hiratsuka S Tomita T Mishima T Matsunaga Y Omori T Ishibashi S. Hepato-entrained B220+CD11c+NK1.1+ cells regulate pre-metastatic niche formation in the lung. EMBO Mol Med. (2018) 10:e8643. doi: 10.15252/emmm.201708643 29930175 PMC6034134

[67] Bubić I Wagner M Krmpotić A Saulig T Kim S Yokoyama WM. Gain of virulence caused by loss of a gene in murine cytomegalovirus. J Virol. (2004) 78:7536–44. doi: 10.1128/JVI.78.14.7536-7544.2004 PMC434107 15220428

[68] Voigt V Forbes CA Tonkin JN Degli-Esposti MA Smith HRC Yokoyama WM. Murine cytomegalovirus m157 mutation and variation leads to immune evasion of natural killer cells. Proc Natl Acad Sci USA. (2003) 100:13483–8. doi: 10.1073/pnas.2233572100 PMC263840 14597723

[69] Tong S Liu G Li M Li X Liu Q Peng H. Natural killer cell activation contributes to hepatitis B viral control in a mouse model. Sci Rep. (2017) 7:314. doi: 10.1038/s41598-017-00387-2 28331190 PMC5428210

[70] Kumar S. Natural killer cell cytotoxicity and its regulation by inhibitory receptors. Immunology. (2018) 154(3):383–93. doi: 10.1111/imm.12921 PMC6002213 29512837

[71] Zompi S Hamerman JA Ogasawara K Schweighoffer E Tybulewicz VLJ Di Santo JP. NKG2D triggers cytotoxicity in mouse NK cells lacking DAP12 or Syk family kinases. Nat Immunol. (2003) 4:565–72. doi: 10.1038/ni930 12740576

[72] Nabekura T Kanaya M Shibuya A Fu G Gascoigne NRJ Lanier LL. Costimulatory molecule DNAM-1 is essential for optimal differentiation of memory natural killer cells during mouse cytomegalovirus infection. Immunity. (2014) 40:225–34. doi: 10.1016/j.immuni.2013.12.011 PMC3943894 24440149

[73] Cifaldi L Doria M Cotugno N Zicari S Cancrini C Palma P. DNAM-1 activating receptor and its ligands: how do viruses affect the NK cell-mediated immune surveillance during the various phases of infection? Int J Mol Sci. (2019) 20(15):3715. doi: 10.3390/ijms20153715 31366013 PMC6695959

[74] Chan CJ Andrews DM McLaughlin NM Yagita H Gilfillan S Colonna M. DNAM-1/CD155 interactions promote cytokine and NK cell-mediated suppression of poorly immunogenic melanoma metastases. J Immunol. (2010) 184:902–11. doi: 10.4049/jimmunol.0903225 20008292

[75] Rocca Y Pouxvielh K Marotel M Benezech S Jaeger B Allatif O. Combinatorial expression of NK cell receptors governs cell subset reactivity and effector functions but not tumor specificity. J Immunol. (2022) 208:1802–12. doi: 10.4049/jimmunol.2100874 35288470

[76] Cossarizza A Chang H-D Radbruch A Abrignani S Addo R Akdis M. Guidelines for the use of flow cytometry and cell sorting in immunological studies (third edition). Eur J Immunol. (2021) 51:2708–3145. doi: 10.1002/eji.202170126 34910301 PMC11115438

[77] Walzer T Bléry M Chaix J Fuseri N Chasson L Robbins SH. Identification, activation, and selective *in vivo* ablation of mouse NK cells via NKp46. Proc Natl Acad Sci USA. (2007) 104:3384–9. doi: 10.1073/pnas.0609692104 PMC1805551 17360655

[78] Carlyle JR Mesci A Ljutic B Belanger S Tai L-H Rousselle E. Molecular and genetic basis for strain-dependent NK1.1 alloreactivity of mouse NK cells. J Immunol. (2006) 176:7511–24. doi: 10.4049/jimmunol.176.12.7511 16751398

